# An Overburdened Ministry

**Published:** 1923-11

**Authors:** 


### HOSPITAL and HEALTH REVIEW

### AN OVERBURDENED MINISTRY.

LAST month we commented upon the many and rapid changes which have taken place in the headship of the Ministry of Health, and made plain our conviction that the department will never thoroughly fulfil the national expectations until greater security of tenure is enjoyed by its paramount chief. The functions of a Ministry of Health have far too intimate a bearing upon the welfare of the people for it to be reasonable that it should be used as a stop-gap for politicians on their promotion. This, important as it is, is, however, by no means all that is to be said on the position of the department, its relations to the country, and its prospects of fulfilling its destiny. That it is an overburdened department is beyond all question, and it can only be a matter of time before this is generally recognised. The matter has, indeed, very recently been raised in a correspondence between Lt.-Col. Fremantle, M.P., and the Prime Minister. Writing in the interests of economy and efficiency the member for the St. Albans Division proposed that the business of housing should be transferred to the Office of Works, and local government to the Home Office, while the Education Office should be suppressed and its functions handed over to the Ministry of Health. The Prime Minister's reply, reduced to a sentence, was that this is not the right moment for change, but that these proposals shall be kept in view when the re-organisation of Government departments enters into the sphere of practical politics.

Although our agreement with Lt.-Col. Fremantle is distinctly limited, we are glad that he has raised the question. The mere fact that he has done so suggests that those who are acquainted with the anomalies of the position realise that, sooner or later, a re-adjustment of the work of certain Ministries will become imperative. Col. Fremantle was formerly Medical Officer of Health for Hertfordshire, and has, no doubt, in that position had opportunities of appreciating the difficulties which weigh upon an office burdened with many incongruous duties, some of which relate only indirectly to health, while others have no relation to it whatever. We fail to see, however, that its duties would be lightened or made more homogeneous by investing it with the functions now discharged by the Education Department. Neither the teaching of the multiplication table, on the one hand, nor the making of grants to secondary schools, on the other, can be said to bear very immediately upon the bodily health of the rising generation. Conversely, we cannot believe that it would be desirable to transfer housing to the Office of Works, a minor department which has little experience of building operations on a large scale. The provision of a sufficiency of well-built and sanitary dwelling-houses is too clearly the business of the Ministry of Health to admit of argument.

When, however, we come to the proposal that local government should be transferred to the Home Office we are on firmer ground. It is ridiculous that the time of the permanent officials of the Ministry of Health—for no one supposes that its head concerns himself personally with such matters—

should be expended upon dealing with the application of Little Pedlington to be allowed to borrow a thousand or two for some purpose entirely alien to the public health. A live Health Department has quite other fish to fry, among them that co-ordination of the health services with which we deal, in a preliminary way, in an article we print this month. This co-ordination is sorely needed and cannot much longer be delayed; but we seriously doubt whether the Ministry of Health, as at present constituted, is in a position to undertake so considerable a task. Its energies are expended, to far too great an extent, upon matters foreign to its *métier*. We have merely to turn over the leaves of Sir George Newman's recent report upon the purely health activities of the great department in Whitehall to be profoundly convinced that those activities, plus the very large matter of housing and slum extinction, are abundantly sufficient to absorb all its energies. The control and prevention of disease are in themselves an undertaking of the first magnitude, and it is impossible to resist the conviction that no Minister can find time or energy to deal effectively with these great matters and at the same time be responsible for the infinite number of questions which arise in connexion with local government property so called. The Ministry of Health was established in a hurry at the end of the war by the joining together of a number of incongruous functions. It is an ill-assorted marriage complicated by the management not of one wife but of many wives, and the sooner there is a multiple process of divorce the better for the Ministry and for the health of the Nation.

